# Fasciolosis: pathogenesis, host-parasite interactions, and implication in vaccine development

**DOI:** 10.3389/fvets.2023.1270064

**Published:** 2023-12-11

**Authors:** Luis Miguel Flores-Velázquez, María Teresa Ruiz-Campillo, Guillem Herrera-Torres, Álvaro Martínez-Moreno, Francisco Javier Martínez-Moreno, Rafael Zafra, Leandro Buffoni, Pablo José Rufino-Moya, Verónica Molina-Hernández, José Pérez

**Affiliations:** ^1^Unidad de Anatomía, Histología y Patología Veterinaria, Escuela de Medicina Veterinaria, Facultad de Ciencias Naturales, Universidad San Sebastián, Campus Puerto Montt, Puerto Montt, Chile; ^2^Departamento de Anatomía y Anatomía Patológica Comparadas y Toxicología, UIC Zoonosis y Enfermedades Emergentes ENZOEM, Universidad de Córdoba, Córdoba, Spain; ^3^Departamento de Sanidad Animal (Área de Parasitología), UIC Zoonosis y Enfermedades Emergentes ENZOEM, Universidad de Córdoba, Córdoba, Spain

**Keywords:** *Fasciola hepatica*, pathogenesis, host-pathogen interaction, immunomodulation, vaccine, livestock, onehealth, zoonosis

## Abstract

*Fasciola hepatica* is distributed worldwide, causing substantial economic losses in the animal husbandry industry. Human fasciolosis is an emerging zoonosis in Andean America, Asia, and Africa. The control of the disease, both in humans and animals, is based on using anthelmintic drugs, which has resulted in increased resistance to the most effective anthelmintics, such as triclabendazole, in many countries. This, together with the concerns about drug residues in food and the environment, has increased the interest in preventive measures such as a vaccine to help control the disease in endemic areas. Despite important efforts over the past two decades and the work carried out with numerous vaccine candidates, none of them has demonstrated consistent and reproducible protection in target species. This is at least in part due to the high immunomodulation capacity of the parasite, making ineffective the host response in susceptible species such as ruminants. It is widely accepted that a deeper knowledge of the host-parasite interactions is needed for a more rational design of vaccine candidates. In recent years, the use of emerging technologies has notably increased the amount of data about these interactions. In the present study, current knowledge of host-parasite interactions and their implication in *Fasciola hepatica* vaccine development is reviewed.

## 1 Introduction

Fasciolosis is a parasitic disease with worldwide distribution, excluding Antarctica. In livestock, it has major economic implications with estimated worldwide economic losses amounting to USD 3,200 million, including anthelmintic treatments, control of intermediate hosts (molluscicides), research, and the implication of economic losses in dairy and meat livestock production (1, 2).

Human fasciolosis has persisted since prehistoric times (3), and currently, it has a significant global health impact in specific geographic locations. The World Health Organization (WHO) has classified fasciolosis as a neglected tropical disease (4), and it is the most geographically distributed parasitic zoonosis (5, 6). *F. hepatica* human infections range between 2.4 and 17 million people (7), with 91 to 180 million people at risk of infection annually (8, 9).

Currently, the control of fasciolosis in ruminants continues to be based on management measures such as pasture rotation and the use of anthelmintics (10). The continued use of anthelmintics has resulted in an increase in parasite-resistant strains for the most effective and widely used flukicides, such as triclabendazole and albendazole (11, 12). Over the past three decades, there has been a rising interest in obtaining vaccines that help prevent and control fasciolosis in ruminants (13). However, the development of vaccines against fasciolosis has been slow, partly due to the great immunomodulatory capacity of the parasite. Hence, a better understanding of the parasite-host interactions is necessary for a more rational design of new vaccine candidates (14, 15).

## 2 Etiology and biological cycle of the parasite

Fasciolosis is caused by flukes of the genus Fasciola, known as liver flukes. The two species most implicated as the etiologic agents of fasciolosis are *F. hepatica*, which is distributed mainly in temperate climate regions, and *F. gigantica*, which is located in tropical regions. Further, hybrid forms have been described in regions where the two species coexist (16, 17). Real-time PCR (qPCR) targeting ITS1 rDNA, ITS2 rDNA, and 28S rDNA have been used to differentiate the two distinct genetic signatures representing each species (18–20). The epidemiological potential of hybridization and introgression between *F. hepatica* and *F. gigantica* remains unknown; therefore, it is important to use the correct terminology consistently and not use the two terms interchangeably (21).

The life cycle of *Fasciola spp*. is quite complex, involving several variations. In general, it involves one or more intermediate hosts, which are the mollusks. At least 20 species of the Lymnaeidae family have been reported as intermediate hosts (22, 23). The asexual larvae undergo several multiplications (24–26) before finally infecting a definitive host in which sexual reproduction occurs.

## 3 Pathogenesis

The penetration, migration, and localization of the parasites in the bile ducts exert a traumatic action that causes a series of lesions in the liver parenchyma and in the bile ducts (27). The newly excysted juveniles (NEJs) of *Fasciola spp*. penetrate the intestinal mucosa and can be found in the abdominal cavity 72 h after metacercaria ingestion. NEJs migrate through the peritoneum to the liver surface and present no clinical sinology in animals (28). The destination of the majority of NEJs is the left hepatic lobe, probably due to its anatomical proximity to the duodenum and the fact that they reach less of the other hepatic lobes. Sometimes, due to massive infestations, these juveniles can have an aberrant migration to other organs, such as the diaphragm and the lung, causing pneumonia and fibrinous pleurisy (29).

Fasciolosis pathogenesis occurs in two phases—the parenchymal and biliary phases. The parenchymal phase begins when the NEJs cross the liver capsule (Glisson's capsule), continuing with the migration of the juvenile stages through the liver parenchyma. This migration causes mechanical damage through abrasion by the tegument that presents spines that help maintain the parasite's position within the liver tissues and probably by-products secreted by migrating larvae. Several pathological processes occur simultaneously within the liver parenchyma, including the migration of juvenile stages that cause necrotic and hemorrhagic lesions, which, in turn, cause inflammatory reactions activating the immune system (30). This response can be found throughout the tortuous migrating trajectory of the parasites, suggesting that the excretion and secretion of these products remain in the tissue, attracting more infiltration of inflammatory cells of an immune nature (31). The biliary phase begins when the parasites enter the bile ducts, where they exert a combined mechanical and chemical action. Through the oral sucker, adult parasites cause mechanical damage while feeding on blood and the liver parenchyma adjacent to the duct. Macerated hepatocytes have been observed inside the sucker and pharynx (27), leading to erosion of the epithelium, trauma, focal rupture of the duct, and puncture of small blood vessels. The enlargement of the bile duct can be chemically induced (32), and it has been suggested that the amino acid proline, which is essential for the synthesis of collagen by fibroblasts, is also released in large quantities by the parasite (33, 34). These two actions exerted by the adult parasite cause a severe eosinophilic and granulomatous inflammatory response, particularly when eggs reach hepatic parenchyma (35), and marked hyperplasia of the bile ducts in which the parasites lodge (36).

The effect of these two phases causes a series of lesions in the liver parenchyma, which is widely correlated with the infective dose; a high dose causes more severe lesions that are more acute and even fatal. However, different studies carried out in sheep (35) and goats (37) have also shown that small repetitive doses (trickle infections) caused more severe hepatic damage than a single dose using the same total number of metacercariae. These findings suggest that the mechanical and enzymatic activities of the parasite may be the initial cause of liver damage. Therefore, the immune response or healing, as well as simultaneous infection at different stages and the immune response to the first infection, play an important role in the pathogenesis of fasciolosis (31).

## 4 Host immune response

### 4.1 Innate immune response

The initial recognition of NEJs takes place within the epithelial mucosa of the intestinal tract with extensive activation. The response to NEJs can occur through the recognition of glycosylated protein and carbohydrate residues that behave as tegumental antigens and induce T-cell proliferation through dendritic cell activation (38, 39). Excretory secretory products containing antigens released by *F. hepatica* (FhESP) can also induce a response of bovine macrophages, which is partially TLR4-dependent (40, 41).

The function of mast cells is not really defined, nor is there evidence that it is protective (42). These cells are residents of tissues that respond to activation of both the innate and acquired immune systems by producing and releasing different inflammatory mediators present in their cytoplasmic granules, prostaglandins, leukotrienes, and certain cytokines such as tumor necrosis factor-alpha (TNF-α) or interleukin-4 (IL-4) (43). In addition, they can release certain active substances against parasites by binding the parasite antigen-IgE complexes with their high-affinity IgE receptors (44, 45). It is estimated that its role is more decisive in the initial stages (peritoneum) of the infection (42, 46, 47). However, it has been described in cattle that after getting infected by *F. hepatica*, there is little evidence of an increase in the percentage of basophils and mast cells (48, 49) and in peritoneal fluid in sheep (50). In contrast, *F. gigantica* infection in buffaloes induces increases in the number of mast cells in the hepatic inflammatory infiltrate (51). In numerous parasitic processes, we can find a population of resident intraepithelial mast cells responsible for rapid parasite rejection phenomena at the epithelial level (52–54). However, these cells have neither been described in the intestine after the migration of *F. hepatica* (30, 36) nor in bile cells such as macrophages and neutrophils, whose function is phagocytic and can release substances such as reagents derived from nitric oxide or active oxygen species that act directly against the parasite (55, 56). On the other hand, infection by *F. hepatica* provokes a Th2-type immune response with IgE production (57) and infiltration of eosinophils and mast cells in the liver (48).

Human neutrophils from patients with acute fasciolosis showed a greater phagocytic function compared to those in the chronic stage of infection (58). Similarly, neutrophils from chronically infected goats showed a poor phagocytic response compared to those from uninfected goats. This poor phagocyte response was correlated with fluke burdens (59). The role of neutrophils in protective responses has not been reported yet in fluke infections.

In cattle, sheep, and goats, *F. hepatica* induces liver and blood eosinophilia, and *F. gigantica* infection in sheep gives the same profile (60–62). However, vaccination of calves and goats showing protection had reduced eosinophil counts (30, 63), which may be due to the lower fluke burdens and hepatic lesions in partially protected animals. In acute stages of *F. hepatica* infection, a dramatic increase of eosinophils has been described in the peritoneal cavity (50, 64) as well as in hepatic lesions, both during the migratory stage (30, 36, 65, 66) and during the chronic stage (35). Eosinophils have been shown to mediate antibody-dependent cell cytotoxicity (ADCC) against *F. hepatica* in rats (42). In Indonesian thin-tailed (ITT) sheep which display resistance to *F. gigantica* but not *F. hepatica*, it has been observed that ADCC by eosinophils plays a role (*ex vivo*) in killing *F. gigantica* but not *F. hepatica* newly excysted juveniles (NEJs) (56). However, peripheral eosinophilia was not related to resistance to *F. gigantica*, suggesting that this cell type is effective only within the gut or peritoneal cavity but not the liver, at least in ITT sheep (67).

Peritoneal macrophages from ITT sheep have also been shown to kill *F. gigantica* but not *F. hepatica* by ADCC (56, 68). This mechanism occurs by attaching effector cells with NEJs in the presence of serum from infected sheep. Macrophages participating in the effective ADCC mechanism against *F. gigantica* showed increased levels of superoxide radicals than those participating in ineffective ADCC against *F. hepatica*, suggesting oxygen radicals play a role in killing *F. gigantica* NEJs (56). It has been reported that in calves protected by experimental vaccination, ADCC mediated by macrophages is nitric oxide-mediated and induces a Th1 cytokine response relying on IgG2a (69). *In vitro* studies have revealed that bovine macrophages were able to kill NEJs in the presence of serum from infected animals. However, NEJs were able to produce molecules such as a family of TGF-like molecules (FhTLM) that significantly reduces ADCC. These macrophages showed features of alternative activation with the expression of high levels of IL-10 (70). In non-protected animals, it has been observed that NEJs induce alternative (M2) activation of macrophages and secrete the regulatory cytokines IL-10 and transforming growth factor-beta (TGF-β) during the peritoneal migration (71–73). M2-activated macrophages have an important role in tissue repair, but they have a reduced capacity to kill NEJs (41, 70).

### 4.2 Adaptive immune response

B-cells have shown importance in *Fasciola spp*.-infected animals as well as in those that have been previously vaccinated (74), highlighting the increase in CD19+ B-cells at the level of hepatic lymph nodes, increasing the recruitment of these cells (66). In cattle, sheep, and goats, IgG1 is the dominant antibody, raising at 4–5 weeks post-infection (wpi) and reaching peaks at 12–15 wpi (37, 75, 76). An increase in specific IgG2 has been shown to correspond to vaccine-induced protection, and an increase in IgG1 has been associated with a non-protective Th2 response (76–78). IgA specific for fluke antigens has not been detected in serum (75), but it has been found in the bile and liver of infected cattle (51), where this immunoglobulin may participate in activating eosinophils to kill NEJs by ADCC (49). Despite this interesting suggestion, few studies have investigated the presence of IgA in bile and liver in both experimental and natural infections.

The immune response exerted during the early stages of fasciolosis is generally regarded as a mixed Th1/Th2 response displaying an increase of certain cytokines such as IFN-γ, IL-4, IL-10, and TGF-β. As the infection progresses, a Th2 response is amplified in conjunction with suppression of Th1 inflammation, thus allowing a prolonged infection that may be dependent on IL-4 (79). In the early stages of sheep and cattle *F. hepatica* infection, both IFN-γ and IL-10 are increased, confirming the initial mixed immune response (75, 80, 81). When the infection progresses, a Th2 response is amplified in conjunction with suppression of Th1 response with reduced IFN-γ and increased IL-4 levels (79). In the early stages of bovine *F. hepatica* infection, both IFN-γ and IL-10 are increased, corroborating the idea that the initial immune response is mixed (75). Buffaloes with both primary and secondary infection of *F. gigantica* also showed a mixed Th1/Th2 response in serum with elevated IFN-γ, IL-4, IL-5, and TGF-β during the early stages of infection. In contrast, when the infection progressed, the Th2 response was dominant (82). The Th1/Th2 response was not the same in different compartments—in sheep liver, IFN-γ increased during the early stages of infection (80, 81), and it remained high during chronic states of infections (81). At the same time, in the hepatic lymph nodes, IFN-γ was reduced both in infected and reinfected animals in acute and chronic stages of infections (81). The high levels of IFN-γ reported in the liver during acute and chronic stages of *F. hepatica* infections contrast with the downregulation of this cytokine in PBMC (83) and hepatic lymph nodes (80, 81) and could be due to a response to hepatic necrosis caused by migrating or adult flukes and granulomata formation.

## 5 Immunomodulation strategies

The inflammatory reaction in fasciolosis is one of the points to be treated primarily to understand the immune response and its evasion. Since metacercariae are excysted in the gut lumen, NEJs are exposed to the host immune response to kill the parasite. However, *Fasciola spp*. has developed a variety of strategies to evade the host response in the different compartments where they stay during the early and late stages of infection, which allows the parasite to live for years within the host. Some of these strategies may be considered passive, as the protection conferred by the tegument, which consists of a syncytial layer covering the entire body of the parasite, formed by a plasma membrane that serves as a support for the outer glycocalyx and a basement membrane that is connected through channels. These structures allow the passage of the components needed for the replacement of the tegument. The rapid replacement of the glycocalyx that covers the tegument—which takes place every 2 to 3 h—may also be an obstacle for products released by inflammatory cells to reach the parasite tegument (84), which is composed of at least 369 proteins. Additionally, the presence of abundant N-glycosylated proteins and glycolipids has made it difficult to characterize its physiological and immune regulatory functions (85).

The majority of strategies used by the parasite to evade the host response may be considered active since they imply the release of a large amount of parasite molecules into the parasite vicinity. These molecules can be released free or within extracellular vesicles (EVs) that are covered by a membrane, and they can be internalized by the host cells, causing their modulation (84, 85). EVs are produced by all developmental stages of *F. hepatica*, and they are considered efficient transporters of parasite molecules to different host compartments, preventing the action of antibodies due to the membrane surrounding the parasite molecules contained in EVs (86). In EVs from *F. hepatica*, up to 618 proteins have been identified, which gives us an idea of how important EVs are for the parasite to interact with the host (87).

*Fasciola spp*. not only use proteins to modulate the host immune response, but EVs also contain microRNAs (miRNAs), molecules with modulating gene expression capacity. miRNAs are abundant in both metacercariae, juvenile and adult *F. hepatica* worms and may play a main role in regulating the developmental and metabolic processes of the parasite, as well as in host-parasite interactions (88–90). The miRNA content in the EVs is different when they are produced by adult or juvenile parasites, leading to different influences in the host cells. These data support the hypothesis that miRNAs are the mediators of the previously demonstrated immune modulatory function of the EVs. However, current data do not allow a fundamental understanding of their regulatory mechanisms in different processes of host-parasite interaction (88–91).

Another mechanism used by liver fluke to survive, migrate, obtain nutrients, and evade the immune response of the host, is the release of excretory secretory products (ESP) (92). FhESP from adult *F. hepatica* contains up to 160 different proteins, including proteases such as cathepsins B and L (FhCB and FhCL), leucine aminopeptidase and carboxypeptidase, fatty acid-binding protein (FABP), and the *F. hepatica* saposin-like protein (FhSAP), all of them necessary for its metabolism (93) (Table 1). FhESP also contains numerous antioxidant enzymes to protect the parasite from reactive oxygen species released by eosinophils and macrophages, such as superoxide dismutase (SOD), glutathione-S-transferase (GST), thioredoxin peroxidase (TPx), and peroxiredoxin (Px) (Table 1). These enzymes not only participate in inactivating reactive oxygen species but also in several important metabolic processes important for parasite survival, such as the excyst of the metacercariae, tissue migration, feeding, and immune evasion (92, 105, 106). Some strategies that *Fasciola spp*. use to evade the host response are discussed below.

**Table 1:** *F. hepatica* molecules involved in host immune modulation/evasion.

**Molecule**	**Actions**	**References**
**Antioxidants:**		
Peroxiredoxins	Antagonizes actions of ROS and induces M2 activation of macrophages	(71)
Thioredoxins			(72)
Glutathione-S- transferase		
Superoxide dismutase		
Glutathione-S-transferase	Induces IL-1β, IL-6, and TNF-α production	(94)
Omega type (GSOT1)	Reduces IL-10 production	
		Induces of macrophage	
**Cysteine proteases**		
Cathepsins L, B	Reduced eosinophils attachment	(95)
Leucine aminopeptidase	Suppression of Th1, Th17	(96)
		Responses, anticoagulants	
**Protease inhibitors:**		
Kunitz type molecule	Suppression of Th1, Th17 responses	(97)
**Other molecules:**		
Fatty binding proteins	Reduction of pro-inflammatory cytokines	(93, 98)
		Induces apoptosis of dendritic cells	(99)
Helminth defense molecule-1	Inhibits APC antigen presentation	(100)
		Inhibits release of IL-1β	
Mucin-like peptides	Increases Th1-type response	(101, 102)
TGF-like molecule	Induces M2-activated macrophages	(70)
Serpin	Prevents the activation of the Lectin complement pathway	(103)
Cystatin	Inhibits NO, IL-6, TNF-α, and promotes the expression of TNF-β and IL-10	(104)
		Induces apoptosis of murine macrophages	(104)

### 5.1 Parasite movement

During the hepatic migration, it has been reported that some larvae show a heavy inflammatory infiltrate, mainly composed of eosinophils attached to the parasite cuticula and in the vicinity of the parasite. However, in other larvae, no inflammatory reaction was found in their vicinity, but necrotic tract and inflammation were observed 2–3 mm behind them (30, 36). It has been suggested that when the parasites are disturbed by the inflammatory reaction, they move ahead, leaving the inflammatory cells behind them (66).

### 5.2 Apoptosis of effector and immune cells

There is an intimate connection between the inflammatory response and the immune response when suffering from fasciolosis. The innate immune response determines the cell populations involved in the inflammatory response by attracting and activating inflammatory cells (107). Eosinophils play a key role in the host response to *Fasciola spp* infection, as suggested by the rapid increase of this cell type in blood, peritoneum, and liver during the early migration of juveniles in sheep (35, 60), cattle (48), and rodents (108). *In vitro* studies have reported that FhESP antigens from *F. hepatica* induce apoptosis of rat eosinophils and macrophages (109, 110). *In vivo* studies have described apoptosis in eosinophils in the liver inflammatory infiltrate during the acute and chronic phases of infection in sheep (65) and the migratory stage in a relevant percentage of peritoneal macrophages, eosinophils, and lymphocytes (50). Increased expression of the pro-apoptotic gene in peripheral blood mononuclear cells of infected sheep and cattle has also been reported (111, 112). More recently, the role of a variety of *F. hepatica* molecules in the induction of apoptosis has been investigated; some of them have been identified as glutathione S-transferase Omega type (GSTO1), which down-regulated the ratio of Bcl-2/Bax and induced increased expression of caspase-3 and apoptosis of macrophages *in vitro* (94). Recombinant cystatin from *F. hepatica* (rFhCystatin) has been shown to induce apoptosis of murine macrophages (104), and fatty acid binding protein (Fh12) induced apoptosis of murine dendritic cells in *in vitro* studies (99).

### 5.3 Modulation of Th1/Th2 and Th17 responses

The immune response mounted during the early stages of fasciolosis is generally a mixed Th1/Th2 response with elevated levels of cytokines such as IFN-γ, IL-4, IL-10, and TGF-β. As the infection progresses, a Th2 response is amplified in conjunction with the suppression of Th1 cytokine production, particularly IFN-γ, which facilitates parasite survival in mice, cattle, and sheep infected with *F. hepatica* (41, 79–81, 113). A similar Th1/Th2 dynamic has been reported in buffaloes infected with *F. gigantica* (82). It has been reported that a variety of parasitic molecules are able to produce modulation of the Th1/Th2 host response; thus, rFhCystatin induced reduced production of IL-6 and TNF-α and increased production of IL-10 and TGF-β in murine macrophages (104). *F. hepatica* Kunitz-type molecule induced suppression of the Th1 and Th17 responses in murine and human dendritic cells (DC) in *in vitro* studies (97).

### 5.4 Modulation of macrophage and antigen-presenting cell functions

In the early stages of *F. hepatica* infection, the recruitment of macrophages and alternative (M2) activation in the peritoneal cavity has been reported in rats at 24 h post-infection (hpi) (71) and at 48 hpi in mice (114). Moreover, FhESP induced M2 activation of peritoneal macrophages in mice (114). In sheep, marked M2 activation has been described by gene expression in PBMC at 7 dpi (83), although peritoneal sheep macrophages showed M2 activation at 24 hpi (73). In cattle, *F. hepatica* also induced M2-activation of macrophages (115, 116). M2-activated macrophages participate in tissue repair, but they show limited ability to control helminth infections (117). *F. hepatica* possesses FhTLM, which is highly expressed in NEJs and unembryonated eggs. It has been reported that FhTLM induces the differentiation of the monocyte-derived macrophages to M2 activation with increased production of IL-10, arginase-1, mannose receptor, and PD-L1 (70).

It has been reported that different antigenic preparations of this parasite, such as total extract, *F. hepatica* tegumental antigen (FhTeg), and *Fasciola hepatica* ESP, decrease the activation state of dendritic cells (DCs) in mice (118–121), and *F. gigantica* ESP induces the modulation of buffalo DCs (122). More specifically, it has been reported that FhTeg induces DC modulation, provoking the absence of T-cell Th1 cytokine response and proliferative activity (38). Glycan products produced by *F. hepatica* have also been reported to induce modulation of DC maturation, resulting in increased production of IL-10 and IL-4 during infection, inducing a Th2/regulatory-polarized immune response (40, 79, 113, 123, 124). In addition, *F. hepatica* cathepsin L1 (FhCL1), glutathione S-transferase (FhGST), and Kunitz-type molecule participate in the modulation of DCs, leading to the suppression of the adaptive immune responses, Th1, and/or Th17 (40, 97). *F. hepatica*-infected sheep showed increased numbers of DCs in the hepatic lymph nodes but reduced expression of MHC class II and CD83, suggesting suppression of the antigen-presentation process in lymphocytes both in the early and late stages of infection (125).

### 5.5 Expansion of T regulatory cells

*F. hepatica*-infected sheep and goats showed expansion of T regulatory cells (Treg) Foxp3+ during early and late stages of infection in the liver and hepatic lymph nodes (50, 81, 126). Moreover, the increase of Foxp3+ cells was more severe in the vicinity of hyperplastic bile ducts during chronic states of infections (50). This expansion of Foxp3+ Treg has been related to IL-10 and parasite survival (127, 128).

## 6 Vaccine development

**Figure 1:**
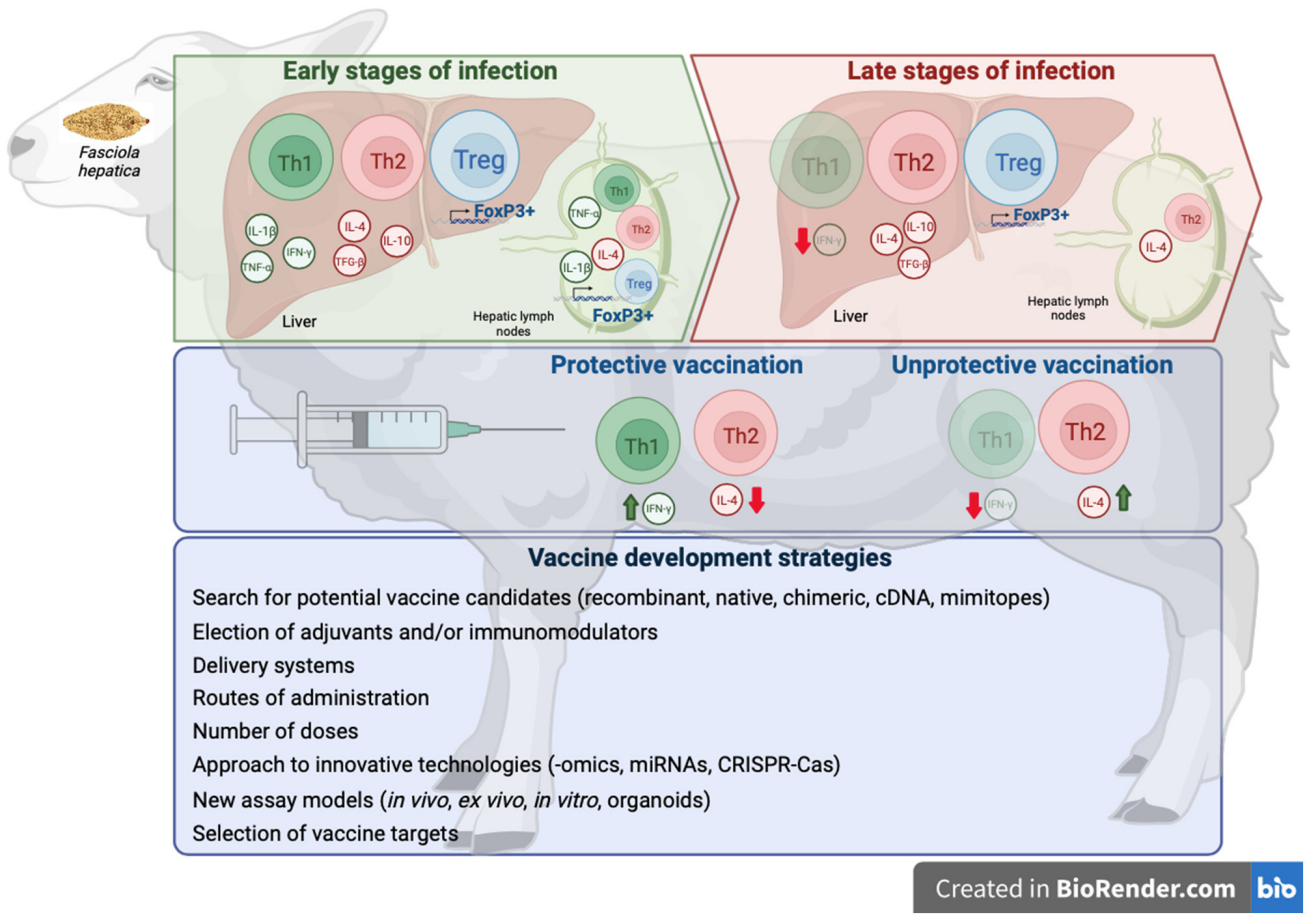
Scheme of immune responses exerted at early and late stages of *F. hepatica* infections, immune responses induced by protective and unproductive vaccines against *F. hepatica*, and strategies to develop effective vaccines. Created with BioRender.com.

Over the past two decades, there have been considerable advances in identifying potential vaccine molecules for the control of fasciolosis in livestock. However, despite some promising results with some vaccine candidates in ruminants, a consistent efficacy required for commercialization has not yet been reached (13). A major obstacle to developing vaccines for fasciolosis is the immune suppression/modulation induced by *Fasciola spp*. that prevents the induction of a protective immune response (Figure 1), evidenced by the lack of immunity observed in naturally and experimentally infected sheep (31, 70, 129). In cattle, natural or experimental infections have been shown to induce certain protection against reinfection, which is maintained long-term (up to 26 weeks post-infection). It has been attributed to the severe fibrosis induced by the primary infection that makes the hepatic migration difficult during the secondary infection (130) or by an increase of intestinal eosinophil and mucosal mast cells (47). Some studies have also reported evidence that protection against *F. hepatica* is inducible in rats, sheep, or cattle by passive transfer of immune sera and cells (131). However, other studies have reported no resistance to reinfection measured by fluke burdens (75). Moreover, no differences in fluke burdens, fecal egg counts, humoral response (specific IgG1 and IgG2), and cell-mediated immune response (IFN-γ production) were reported in calves challenged with *F. hepatica* after single or trickle infection (48, 57, 75) suggesting that reinfections do not induce protection. Experimental studies reported no protection against reinfection in sheep (35, 81, 132) and goats (37), although the host response was different; thus, primo-infected sheep showed a mixed Th1/Th2/Th17 response while reinfected ones presented a more Th2 polarized response (81) and a lower humoral response (132).

It has been reported that in protective vaccines in sheep (133) and goats (134), a mixed Th1/Th2 response was found with higher levels of IFN-γ and lower levels of IL-4 in vaccinated groups than in the infected control group (133). In sheep immunized with a non-protective vaccine, the host immune response showed a predominantly Th2 profile during chronic stages of the infection, similar to that found in non-vaccinated and infected animals (80). The challenge is to identify the specific antigens that are the targets of this protective immunity and incorporate these in vaccine formulations that induce a mixed Th1/Th2 response to enhance vaccine efficacy (135). It has been estimated that a vaccine with an efficacy of 50–60% in fluke reduction would likely be beneficial in numerous countries to significantly reduce economic losses, and it also would have a positive impact on epidemiology by reducing eggs in pasture (13).

Several strategies have been used to design vaccine candidates for fasciolosis in livestock. The first vaccine trials used native proteins isolated using conventional biochemical methods from the excreted/secreted (ES) proteins of adult parasites (136, 137). Despite good protection being found in sheep and cattle in these trials using native FhCL1 and FhGST, the use of native proteins in a commercial vaccine for fasciolosis in livestock is not feasible, which is why the majority of subsequent vaccine trials have been carried out using recombinant proteins of different stages of the parasite (13). Some vaccine trials using recombinant proteins reported high protection of up to 89% in fluke reduction (Tables 2, 3); however, this high protection has not been reproducible in different labs and conditions. A combination of recombinant vaccines (cocktail vaccines) has also been used recently with variable efficacy (Table 3). The majority of vaccine trials have used the subcutaneous or intramuscular administration route. However, a few trials have used mucosal vaccine delivery with promising results. For instance, Norbury et al. (154) administered a cocktail vaccine containing FhCL5 and FhCB2 by an intranasal method in sheep, obtaining a 40.5% fluke reduction and a 92% egg viability reduction, while the same vaccine administered intramuscularly did not induce protection. The oral route has also been used to administer freeze-dried transgenic lettuce expressing the cysteine proteinase of *F. hepatica* (CPFhW) in sheep and cattle, inducing significant protection in cattle (56.2%) and 35.5% fluke reduction (not significant) in sheep (142).

**Table 2:** Summary of fasciolosis single vaccines in livestock.

**Antigen (μg per dose)**	**Species (sex_age)/No. per group**	**Admin. Route (no. doses)/time**	**Adjuvant**	**Efficacy^†^**	**References**
**Cathepsin L**					
rFhCL1 (200)	Cattle (m_3-8mo.)/13	s.c.(2)/3w	Montanide^TM^ ISA 70VG or 206VG	48%	(69)
rFhCL1 (100)	Goat (m_4mo.)/10	s.c.(2)/4w	QuilA	ns	(30)
rFhpCL1 (100)	Sheep (f_4-6mo.)/5	s.c.(2)/4w	QuilA	ns	(138)
CL1 mimitopes (§)	Sheep (nd_9mo.)/5	s.c.(2)/2w	None	51%	(139)
CL1 mimitopes (§)	Sheep (m_9mo.)/5	s.c.(2)/4w	QuilA	57.5%	(140)
CL2 mimitopes (§)	Sheep (m_9mo.)/5	s.c.(2)/4w	QuilA	ns	(140)
CL1 mimitopes (§)	Goat (m_9mo.)/5	s.c.(2)/4w	QuilA	55.4%	(141)
CL1 mimitopes (§)	Goat (m_9mo.)/5	s.c.(2)/4w	QuilA	70.4%	(141)
CL2 mimitopes (§)	Goat (m_9mo.)/5	s.c.(2)/4w	QuilA	ns	(141)
CL1 mimitopes (§)	Goat (nd_6mo.)/6	s.c.(2)/4w	QuilA	46.9-79.5%	(134)
**Cathepsin**					
rCPFhW (300)	Sheep (m&f_5mo.)/6	oral(2)/4w	None	35.5%	(142)
rCPFhW (500)	Cattle (m&f_5-7mo.)/6	oral(2)/4w	None	56.2%	(142)
**Leucine Amino-Peptidase (LAP)**					
rFhLAP (100)	Sheep (m_12mo.)/10	s.c.(2)/4w	FCA/FIA, Adyuvac 50, Alum, DEAE-D, or Ribi	49–89%	(143)
rFgLAP (150&300)	Buffalo (nd_8-10mo.)/7	i.m.(3)/3w	Montanide^TM^ M-70 VG	ns	(144)
**Fatty acid binding protein (FABP)**					
rFh15 (150)	Sheep (nd_nd)/6	s.c.(2)/5d	ADAD (Qs, PAL, Montanide^TM^ ISA763A)	43%	(145)
rFgFABP (400)	Buffalo (nd_8-10 mo.)/5	s.c.(3)/3w	FCA/FIA	35%	(146)
rFgFABP (400)	Buffalo (nd_8-10 mo.)/7	i.m.(3)/3w	Montanide^TM^ M-70 VG	ns	(147)
rSm14 (100)	Goat (m_6mo.)/7	s.c.(2)/4w	QuilA	ns	(148)
**Glutathione S transferase**					
rFgGST (400)	Buffalo (nd_8-10 mo.)/7	i.m.(3)/3w	Montanide^TM^ M-70 VG	ns	(147)
rFhGST (100)	Goat (m_4mo.)/10	s.c.(2)/4w	QuilA	ns	(36)
**Helminth defense molecule**					
sMF6p/FhHDM1 (100)	Sheep (f_4-6mo.)/5	s.c.(2)/4w	QuilA	6%	(138)
nMF6p/FhHDM1 (100)	Sheep (f_4-6mo.)/5	s.c.(2)/4w	QuilA	15%	(138)
**Thioredoxin**					
rFhTGR (300)	Cattle (nd_nd)/8	s.c.(3)/4w	FIA	8.2%	(149)
rFhTGR (400)	Cattle (nd_nd)/6	s.c.(2)/4w	Adyuvac50	3.8%	(149)
rFhTGR (400)	Cattle (nd_nd)/6	s.c.(2)/4w	Alum	23%	(149)
**Glutathione reductase phospho-glicerate kinase**					
cFhPGK/pCMV (100)	Sheep (m_5mo.)/8	i.m. (3)/4w	Montanide^TM^ ISA 206	ns	(150)
cFhPGK/pCMV (100)	Sheep (m_5mo.)/6	i.m. (3)/4w	CTLA-4	ns	(150)
**14-3-3z**					
r14-3-3z (100)	Sheep (f_6mo.)/8	s.c.(2)/4w	Montanide^TM^ ISA 71 VG	ns	(151)
**Tetraspanin**					
rFhTSP2 (200)	Cattle (f_6mo.)/6	s.c.(2)/4w	FCA/FIA	ns	(152)

^†^percentage expressing only significant efficacy; §1 × 10^13^ phage particles; ADAD, Adaptation adjuvant (ADAD) system; c, cDNA; d, days; DEAE-D, Diethylaminoethyl-dextran; f, female; FCA/FIA, Freund's complete adjuvant and Freund's incomplete adjuvant; Fh, *Fasciola hepatica*; Fg, *Fasciola gigantica*; i.m., intramuscular; m, male; mo., months; n, native; nd, not defined; ns, non-significant; PAL, the hydroalcoholic extract of *P. leucotomos*; Qs, saponin from *Q. saponaria*; r, recombinant; Ribi, MPL + TDM + CWS Adjuvant System (Sigma–Aldrich); s, synthetic; s.c., subcutaneous; w, weeks apart between doses.

**Table 3:** Summary of fasciolosis combined vaccines in livestock.

**Antigens (μg each per dose)**	**Species (sex_age)/No. per group**	**Admin. Route (no. doses)/time**	**Adjuvant**	**Efficacy^†^**	**References**
CL1 + CL2 mimitopes (§)	Sheep (m_9mo.)/5	s.c.(2)/4w	QuilA	ns	(140)
CL1 + CL2 mimitopes (§)	Goat (m_9mo.)/5	s.c.(2)/4w	QuilA	32.4%	(141)
rmFhCL1 + rmFhCL3 (200)	Cattle (m_6-8mo.)/5	s.c.(2)/3w	ZA1	37.6%	(153)
rmFhCL1 + rmFhCL3 (200)	Cattle (m_5-11mo.)/5	s.c.(2)/2w	ZA1	ns	(153)
rCatL5 + rCatB2 (150)	Sheep (m_5mo.)/8	i.m.(3)/4w	QuilA	20.9%	(154)
rCatL5 + rCatB2 (75)	Sheep (m_5mo.)/8	i.n.(3)/4w	CpG-ODN + ISC-adjuvant	40.5%	(154)
rFhLAP + chCL1(100)	Sheep (m_8mo.)/5	s.c.(2)/2w	QuilA	25.5%	(155)
rFhLAP + chCL1(200)	Sheep (m_8mo.)/5	s.c.(2)/2w	QuilA	30.7%	(155)
rFhLAP + chCL1(400)	Sheep (m_8mo.)/5	s.c.(2)/2w	QuilA	40.6%	(155)
rFhTeg1 + rFhTeg5 (200)	Cattle (f_6mo.)/7	nd(2)/4w	FCA/FIA	ns	(156)
rFhCL1 + rFhHDM + rFhLAP + rFhPrx (100)	Sheep (m_8mo.)/10	s.c.(2)/4w	Montanide^TM^ ISA 61	37.2%	(157)
rFhCL1 + rFhHDM + rFhLAP + rFhPrx (100)	Sheep (m_8mo.)/10	s.c.(2)/4w	Alum	ns	(157)
rFhStf1 + rFhStf2 + rFhStf3 + rFhKT1 (100)	Sheep (f&m_8mo.)/14	s.c.(3)/3w	Montanide^TM^ ISA 61	17.4%	(15)
rFhStf1 + rFhStf2 + rFhStf3 + rFhKT1 (100)	Sheep (m_8mo.)/13	s.c.(3)/3w	Montanide^TM^ ISA 61+CpG	0%	(15)
rLTB-rFhTSP2 (451)	Cattle (f_6mo.)/6	i.n.(2)/4w	None	ns	(152)

^†^percentage expressing only significant efficacy; §1 × 10^13^ phage particles; CatL5, Cathepsin L5; CatB2, Cathepsin B2; CL1, Cathepsin L1; CL2, Cathepsin L2; CpG-ODN, CpG-oligodeoxynucleotide; ch, chimeric; d, days; f, female; FCA/FIA, Freund's complete adjuvant and Freund's incomplete adjuvant; Fh, *Fasciola hepatica*; HDM, helminth defense molecule; i.m., intramuscular; i.n., intranasal; ISC-adjuvant by Zoetis; KT1, K unit 1; LAP, Leucin aminopeptidase; LTB, Heat labile enterotoxin B subunit; m, male; mo., months; nd, not defined; ns, non-significant; Prx, Peroxiredoxin; r, recombinant; rm, recombinant mutant; s, synthetic; s.c., subcutaneous; Stf, Stefin; Teg1, Tegumental glycoprotein 1; Teg5, Tegumental glycoprotein 5; TSP2, Tretaspanin 2; w, weeks apart between doses.

Most vaccine trials in ruminants have used proteases, antioxidant enzymes, or fatty acid-binding proteins as antigens (Tables 2, 3). However, these proteins are quite abundant in *Fasciola* spp, and blocking one or several of them by a vaccine probably does not cause serious problems to the worm since it has other proteins with similar functions. This might be a reason for the limited efficacy obtained in the numerous vaccine trials conducted with these antigens in ruminants.

## 7 Conclusion and remarks

The slow progress to date in developing a protective vaccine to be used in the control of fasciolosis in livestock suggests that new approaches should be investigated, such as the use of new antigens, evaluation of immunity induced by recombinant proteins, use of different adjuvants, formulations, and delivery systems. Despite important advances in the knowledge of host-parasite interactions in fasciolosis, a more rational vaccine candidate design requires a deeper knowledge of the mechanisms and molecules involved in host-parasite cross-talk in relevant target host species (sheep, cattle, goats, buffalo). The progress of the -omics technologies and the immunoinformatic/immunoproteomic approaches should provide useful data in the next few years. An example is the new proteomic technologies applied to NEJs after crossing the gut (158) or during the early stages of hepatic migration, which may be useful to select new vaccine candidates directed against NEJs, a stage of the parasite that it is more exposed to the host immune system than adult ones located within the bile ducts.

## Author contributions

LF-V: Writing—original draft. MR-C: Writing—original draft. GH-T: Writing—review & editing. ÁM-M: Writing—review & editing. FM-M: Writing—review & editing. RZ: Writing—review & editing. LB: Writing—review & editing. PR-M: Writing—review & editing. VM-H: Conceptualization, Writing—original draft, Writing—review & editing. JP: Conceptualization, Writing—original draft, Writing—review & editing.
